# Editorial: Immunopathology of Type 1 Diabetes

**DOI:** 10.3389/fimmu.2022.852963

**Published:** 2022-03-10

**Authors:** E. Allison Green, Anne C. Cooke, Jon D. Piganelli, Sarah J. Richardson, Li Wen, F. Susan Wong

**Affiliations:** ^1^ Hull York Medical School, University of York, York, United Kingdom; ^2^ Department of Pathology, University of Cambridge, Cambridge, United Kingdom; ^3^ Department of Pediatric Surgery, Rangos Research Center, UPMC Children’s Hospital of Pittsburgh, University of Pittsburgh, Pittsburgh, PA, United States; ^4^ Islet Biology Group, Exeter Centre for Excellence in Diabetes (EXCEED), Institute of Biomedical and Clinical Sciences (IBCS), University of Exeter, Exeter, United Kingdom; ^5^ Section of Endocrinology, Department of Internal Medicine, Yale University School of Medicine, New Haven, CT, United States; ^6^ Division of Infection and Immunity, Cardiff University School of Medicine, Cardiff University, Cardiff, United Kingdom

**Keywords:** type 1 diabetes, autoimmunity, NOD mouse model, adaptive immunity, innate immunity

Our understanding of the islet-immune interface in autoimmune diabetes has exploded with the vast array of knowledge coming from recent works. Historically we know that genetic susceptibility and environmental influences trigger, or perpetuate gradual autoimmune destruction and damage to the insulin-producing islet beta (β) cells. However, the events leading to the breakdown in tolerance towards islet β cells, and the ensuing pro-inflammatory cytokine laden environment that perpetuates T cell-mediated β cell destruction, remain elusive.

This timely Research Topic comprises a compendium of 5 original research articles and 15 reviews focusing on recent advances in the immunology of type 1 diabetes (T1D) that build on earlier observations and provide new knowledge. These articles center on environmental drivers of β cell destruction and use new models and technologies to interrogate factors influencing the breakdown of self-tolerance, enhancing our understanding of T1D immunopathology.

In a thorough review, Quinn et al. describe data from past and present clinical trials that have assessed environmental elements associated with progression to T1D. The authors examined the likelihood of true causality of many favored initiators (beyond the well-characterized genetic predisposition, primarily at the HLA loci) activating self-reactive T cells that target β cells, in this heterogeneous condition. Environmental determinants, including enterovirus infection, rapid weight gain in early life, and the microbiome, correlated highly with T1D incidence, suggesting a ‘threshold hypothesis’ where genetic and environmental factors interact to promote T1D over time. The pancreatropic viruses, particularly the T1D-associated coxsackievirus B (CVB), are prominent contenders. In support of the viral induction hypothesis, Morse and Horwitz provide a compelling review describing how the antiviral response can modulate the microbiome, causing dysbiosis, and diabetes onset. This work stresses the importance of communication between the intestinal microbiota and the local immune population in dictating the outcome of the interaction. This interaction is also influenced by other predisposing factors, such as genetic predisposition, viral responses leading to dysbiosis and the background state of the host immune system. Further, Lincez et al. used elegant animal models that express altered expression of two key viral sensors-melanoma differentiation-associated protein 5 (MDA5) and toll-like receptor-3 (TLR-3). They showed that alterations in sensing of the same virus (CVB) by MDA5 and TLR3 led to unique IFN-α and IFN-β signatures, which profoundly affected disease outcomes. Specifically, infection with islet β cell-tropic CVB4, under reduced MDA5 signaling, protected against diabetes, whereas reduced TLR3 function did not influence diabetes susceptibility. Thus, the pressure of a diabetogenic β cell-tropic virus, in conjunction with a genetic predisposition to autoimmunity, creates a perfect storm, providing the necessary inflammatory signature leading to disease onset. Interestingly, Zipris presents an alternative concept linking viruses to T1D, involving visceral adipose tissue. Zipris discusses how the Kilham Rat Virus, which causes diabetes in BioBreeding and LEW1.WR1 rats, also induces inflammation in visceral adipose tissue (VAT). Infection is associated with macrophage recruitment, pro-inflammatory cytokine and chemokine upregulation, as well as endoplasmic reticulum (ER) and oxidative stress responses. Together, these have a negative impact on insulin signaling, and may link to autoimmune diabetes progression.

However, β cells are more than innocent bystanders in the progression to diabetes. Toren et al. provide evidence that the ER in β cells is under tremendous strain to maintain euglycemia. When this metabolic demand is coupled with the highly vascularized setting needed for insulin uptake into the bloodstream, pathology may result. β cells exposed to noxious agents including pro-inflammatory cytokines and chemokines, and immune cells that extravasate into the islet, will respond to pathogen-associated molecular patterns (PAMPs) by releasing danger-associated molecular patterns (DAMPs). These, in turn, mobilize innate and adaptive immune cells to the target tissue, triggering β cell death. Thus, a focus on the role of the β cell as an active participant, rather than an unfortunate victim in T1D progression, may allow us to identify new targets in T1D. In this context, Ding et al. focus on the role of alpha-protein kinase 1 (ALPK1), a newly identified cytosolic pathogen-recognition receptor (PRR) specific for ADP-β-D-manno-heptose (ADP-heptose) associated with β cells, and how it predisposes β cells to cytokine-mediated apoptosis *via* upregulation of the TNF-α signaling pathway. Using the Min6 murine β cell line, mechanistic investigations showed that ALPK1 activation was sufficient to induce the expression of TNF-α and Fas after cytokine stimulation. It will be interesting to determine whether this translates to primary beta cells, establishing the *in vivo* significance of these findings. The PAMP ADP-heptose may also arise in the islet *via* the dysbiosis described by Morse and Horwitz, leading to the exacerbation of cytokine signaling seen in β cells.

The cells and molecules of innate immunity involved in the early T1D responses, particularly the inflammasome, have been discussed by Pearson et al. They highlight altered gut microbial composition and associated influences on inflammasome activity and T1D development. As modulation of inflammasomes has had some therapeutic success in other autoimmune diseases, a similar approach may have clinical benefits for T1D. Focusing also on innate immunity, Klocperk et al., studying children with T1D and first-degree relatives, showed that neutrophilia and neutrophil products including neutrophil extracellular traps (NETs), myeloperoxidase (MPO), neutrophil elastase (NE), proteinase 3 (PR3) and LL37, were evident before diabetes onset, but reduced over time. In addition, Huang et al. found that IL-10 deficiency in diabetogenic T cell receptor (BDC2.5) transgenic mice promoted the expansion of bone marrow and peripheral neutrophils. IL-10 deficiency enhanced neutrophil expression of IFNγ and IL-1β compared with IL-10-sufficient controls. IL-10 plays an important regulatory role systemically and in mucosal immunity, and IL-10-deficient BDC2.5 NOD mice had altered gut microbiota, which in turn modulated systemic neutrophil homeostasis. The innate lymphoid cells (ILC), another group of innate cells, classified by their cytokines and transcription factor profiles, may be considered to be the innate counterparts of the T helper subsets. Stojanovic et al. review gut-associated lymphoid tissue residing ILC3, which secrete IL-17 and GMCSF, in T1D development and therapeutic targeting. Finally, Gardner and Fraker comprehensively review the role of innate NK cells in immunopathogenesis of T1D development. β cells express NK ligand(s), which may contribute to their direct killing by NK cells. It is noteworthy that specific NK cell markers, such as NKG2D, are also expressed on CD8^+^ T cells, especially human CD8^+^ T cells, which are central adaptive immune cell players in β-cell destruction.

Adaptive immunity, focused on both effector and regulatory cells (Tregs), has long been implicated in the pathogenesis of T1D. Foxp3-expressing Tregs regulate autoimmune disease; humans with a mutated *FOXP3* gene develop the multiorgan autoimmune syndrome. Here, Watts et al. utilized DEREG (Depletion of REGulatory T cells) mice where a bacterial artificial chromosome (BAC)-encoded Foxp3 promoter controls fluorescent diphtheria toxin (DTR-eGFP) fusion protein expression. Using diphtheria toxin-mediated transient Treg depletion, in NOD mice, they found that T1D was induced only in the mice where pancreatic infiltration was already present. Furthermore, Treg depletion exacerbated a Th1 type response in the pancreas and associated lymph nodes, highlighting the importance of CD8+ T cells as effectors of autoimmune-mediated beta cell destruction.

Advances in techniques that can investigate the transcriptome, epigenetic and proteomic profile at the single-cell level are revolutionizing our understanding of the immune system in health and disease. In Bode et al., the history of approaches taken to assess global immune cell gene changes and the emergence of single-cell studies that assess the heterogeneity of transcriptome, epigenetics and proteasome in immune cells is reviewed. Using cancer immunology as exemplars, single-cell approaches are adaptable to investigate facets of T1D. Hanna et al. extend this theme, reviewing single-cell RNAseq studies from human T1D. RNAseq analysis of human immune cells from various tissues has facilitated novel discoveries of the cytokine profiles and exclusive gene expression that may be predictive of T1D progression. However, the use of scRNAseq is not without challenges, requiring robust computational approaches to facilitate biomarker discovery. There is a clear need for biomarkers in T1D to track progression and monitor the efficacy of therapeutic interventions, particularly T cell-based biomarkers, given that many current immunotherapies for T1D target T cells. Nakayama and Michels review the current knowledge and potential for using the T cell receptor (TCR) as a biomarker in T1D. They focused on pancreatic infiltrating T cells and revealed proinsulin or insulin reactivity. They suggested that these cells were more reactive or had higher affinity TCRs than comparable cells in the peripheral blood. Documenting the challenges of current technical approaches to identify unique clusters of TCRs associated with T1D, newer approaches using high throughput sequencing of tens of millions of TCR clonotypes are proving more insightful. Nevertheless, the robustness of using TCR clonotypes as biomarkers will need TCR datasets from many individuals with and without T1D to elicit the best performance by machine learning and clustering algorithms.

B cells also play an important role in the pathogenesis of T1D, beyond their function as antibody-producing cells. In a review by Greaves et al., a detailed discussion of the development, phenotype and function of enigmatic thymic B cells in health and T1D is provided. Interestingly, the emergence of thymic ectopic germinal centers is a commonality in lupus, myasthenia gravis and T1D, all diseases where thymic B cells may have a proposed pathogenic role. Studying the human thymus is difficult, and the authors discuss the potential of computational modeling to evaluate key pathways by which thymic B cells may perturb central T cell tolerance. Like T cell responses, there is heterogeneity in B cell responses. Boldison and Wong have reviewed the role in T1D of distinct subsets of regulatory B cells in mice and man, highlighting their cytokine profiles, costimulatory requirements, and interactions with the innate immune system to elicit their function. In human T1D, the definitive role for regulatory B cells is controversial. Future studies on heterogeneity of regulatory B cell repertoires at defined stages of T1D and dissection of their cross-talk with other immune cells will help resolve this debate.

To what are the T cells responding? Various antigens, suggested by antibody reactivities, have been identified as targets for the autoreactive T cells in diabetes. Whilst for both humans and mice, peptides of proinsulin are targets for both CD8^+^ and CD4^+^ T cells; however, there are still some intriguing biological questions. Jhala et al., building on a previous observation that proinsulin-1 deficient mice are protected from autoimmune diabetes, have further investigated autoimmune responses to proinsulin-1, using a mouse model with tetracycline-regulated expression of proinsulin-1 in antigen-presenting cells (TIP-1 mice). They found that the mice had reduced proinsulin-1-specific T cells, reduced insulitis and diabetes, and the proinsulin-1 specific cells were less able to transfer diabetes to an immunodeficient recipient, all of which indicate the induction of immune tolerance. Post-translational modifications of self-proteins, such as the post-translational conversion of arginine to citrulline residues by peptidylarginine deiminase enzymes can also produce novel T cell targets in T1D. Such modified antigens would not be encountered in the thymus by developing T cells, and thus central tolerance to them would not occur. The mini-review by Reed and Kappler documents how epitopes of unique chimeric antigens are generated post-translationally following β cell-granule fusion with lysosomes, in a process called crinophagy. Secreted exosomes transport the chimeric peptides as cargo to draining lymph nodes where CD4^+^T cell stimulation of diabetogenic T cells could occur. However, the key activation signal for these strongly stimulatory peptides is not clear and remains an important further question for consideration.

Finally, Armitage et al. provide an in-depth review on the role and function of the protein tyrosine phosphatase, non-receptor type 22 (PTPN22), a negative regulator of T and B cell receptor signaling. This review describes an expanded role for this phosphatase in controlling many cells in the immune system. It could explain why dysregulation of this molecule can profoundly impact normal immune function. The authors also describe a set of single nucleotide polymorphisms (SNPs) at the *PTPN22* locus, leading to immune defects that precipitate the loss of self-tolerance and progression to autoimmune diabetes. Since the SNP in PTPN22 (rs2476601) is associated with TCR and BCR signaling and other adaptive and innate immune cell processes, it is of major interest to further define the downstream effects of suboptimal control of its signaling in people living with T1D.

Overall, the studies outlined in this Research Topic highlight the heterogeneity of factors that may lead to the development of T1D. There is a critical need for a deeper understanding of these factors if we are to develop new immunotherapeutic strategies, which may need to be multifaceted to be most effective for both prevention, and as a therapeutic in those in whom diabetes has already developed.

## Author Contributions

All authors wrote the manuscript and edited the final version.

## Conflict of Interest

The authors declare that the research was conducted in the absence of any commercial or financial relationships that could be construed as a potential conflict of interest.

## Publisher’s Note

All claims expressed in this article are solely those of the authors and do not necessarily represent those of their affiliated organizations, or those of the publisher, the editors and the reviewers. Any product that may be evaluated in this article, or claim that may be made by its manufacturer, is not guaranteed or endorsed by the publisher.

